# Patients’ Perspectives on Commencing Oral Anticoagulants in Atrial Fibrillation: An Exploratory Qualitative Descriptive Study

**DOI:** 10.3390/pharmacy11050153

**Published:** 2023-09-23

**Authors:** Eyob Alemayehu Gebreyohannes, Sandra M. Salter, Leanne Chalmers, Jan Radford, Kenneth Lee, Danielle D’Lima

**Affiliations:** 1 Division of Pharmacy, School of Allied Health, The University of Western Australia, Crawley 6009, WA, Australiakenneth.lee@uwa.edu.au (K.L.); 2 Curtin Medical School, Curtin University, Bentley 6102, WA, Australia; 3 Launceston Clinical School, Tasmanian School of Medicine, University of Tasmania, Launceston 7250, TS, Australia; j.radford@utas.edu.au; 4 Centre for Behaviour Change, Department of Clinical, Educational and Health Psychology, University College London, London 181445, UK

**Keywords:** atrial fibrillation, oral anticoagulants, treatment refusal, primary health care, behavioural change wheel, theoretical domains framework, qualitative research

## Abstract

Background: Oral anticoagulants (OACs) are prescribed to patients with atrial fibrillation (AF) in order to lower stroke risk. However, patient refusal to commence OACs hinders effective anticoagulation. This study aimed to explore barriers and facilitators to patient agreement to commence OACs from the perspectives of patients with AF attending Australian general practices. Methods: A qualitative descriptive study utilising semi-structured individual interviews was conducted from March to July 2022. Results: Ten patients (60% male, median age = 78.5 years) completed interviews. Patients’ passive roles in decision-making were identified as a facilitator. Other prominent facilitators included doctors explaining adequately and aligning their recommendations with patients’ overall health goals, including the prevention of stroke and associated disabilities, and a clear understanding of the pros and cons of taking OACs. Reportedly insufficient explanation from doctors and the inconvenience associated with taking warfarin were identified as potential barriers. Conclusion: Addressing factors that influence patient agreement to commence OACs should be an essential aspect of quality improvement interventions. Subsequent studies should also delve into the perspectives of eligible patients with AF who choose not to commence OACs as well as the perspectives of both patients and doctors regarding the decision to continue OAC treatment.

## 1. Introduction

Atrial fibrillation (AF) is the most common arrhythmia in clinical practice and affects an estimated 60 million people in the world [1]. AF is known to increase the risk of stroke five-fold [2]. To prevent stroke in patients with AF, oral anticoagulants (OACs) are recommended, as they are associated with a 70% relative risk reduction [3]. OACs include vitamin K antagonists such as warfarin and direct oral anticoagulants (DOACs) such as apixaban, rivaroxaban, and dabigatran [3]. DOACs and warfarin account for 76% and 24% of prescribed OACs, respectively, among patients with AF in the Australian general practice setting [4]. Major contemporary AF guidelines such as those from the 2020 European Society of Cardiology and the 2018 Australian AF guidelines recommend OACs for patients with AF at high stroke risk, as determined by a CHA_2_DS_2_-VA score (i.e., the sex-less CHA_2_DS_2_-VA score) of ≥2. They also suggest considering OACs in patients at moderate risk (CHA_2_DS_2_-VA score of 1) [2,3]. Current evidence suggests that prescribing OACs in accordance with guideline recommendations (i.e., guideline adherence) is linked to enhanced clinical outcomes in patients with AF [5,6]. However, it is commonly observed that guideline non-adherence is prevalent worldwide, including within the primary care setting [7]. Guideline non-adherence primarily relates to OAC non-prescription in patients with AF who are at high risk of stroke, which could be because of doctor- or patient-related factors [7].

General practitioners (GPs), also referred to as primary care physicians or family physicians, serve as the primary healthcare providers and the initial access point to the healthcare system in many countries [8]. In countries like Canada, France, and Australia, they represent approximately half of the total doctor workforce [9]. In Australia, primary care management of chronic conditions is typically provided by GPs, and AF is among the most frequently managed chronic conditions in general practice [10,11]. However, recent studies showed that 35–45% of patients who were at high risk of stroke and attending Australian general practices did not receive an OAC prescription, suggesting a need for improvements [4,12].

In a recent exploratory qualitative study of nine GPs conducted in Australia, GPs reported that patient refusal to commence OACs contributed to guideline non-adherence in the management of patients with AF [13]. Patient refusal to commence OACs is also among the most frequently reported reasons in the literature leading to guideline non-adherence [14,15,16,17,18,19,20,21]. Although many previous studies mentioned patient refusal as a reason for OAC non-prescription [14,15,17,18,20,21], the underlying reasons for refusal were not investigated in these studies. In addition, most studies did not include patient perspectives [15,16,17,18,19,21] and focused only on vitamin K antagonists [15,17,18,20], but vitamin K antagonists now represent less than a quarter of the prescriptions given to patients with AF in the Australian general practice setting [4]. Most studies were also conducted outside Australia [14,16,17,18,19,20] and/or included only hospitalised patients with a specific concomitant disease [22]. Patient agreement to commence OACs is a behaviour, which can be better understood by drawing upon theoretical frameworks or models from the field of behaviour change science. The application of such theoretical frameworks or models enables a thorough and theory-informed exploration of key influences on a behaviour, which can be used to develop systematic interventions to support change [23,24]. The Theoretical Domains Framework (TDF) [23] and the Capability, Opportunity, Motivation—Behaviour (COM-B) model [24] are commonly used to identify key influences on behaviour [25].

### 1.1. Aims

The main aim of this study was to explore patients’ perspectives on OAC initiation with the goal of identifying key influences on behaviour. Specifically, we aimed to explore barriers and facilitators to patient agreement to commence OACs from the perspectives of patients with AF attending Australian general practices.

### 1.2. Ethics Approval

Ethics approval was obtained from the University of Western Australia Human Research Ethics Committee on 26 November 2021 (2021/ET000999).

## 2. Materials and Methods

### 2.1. Study Design

**Figure 1:**
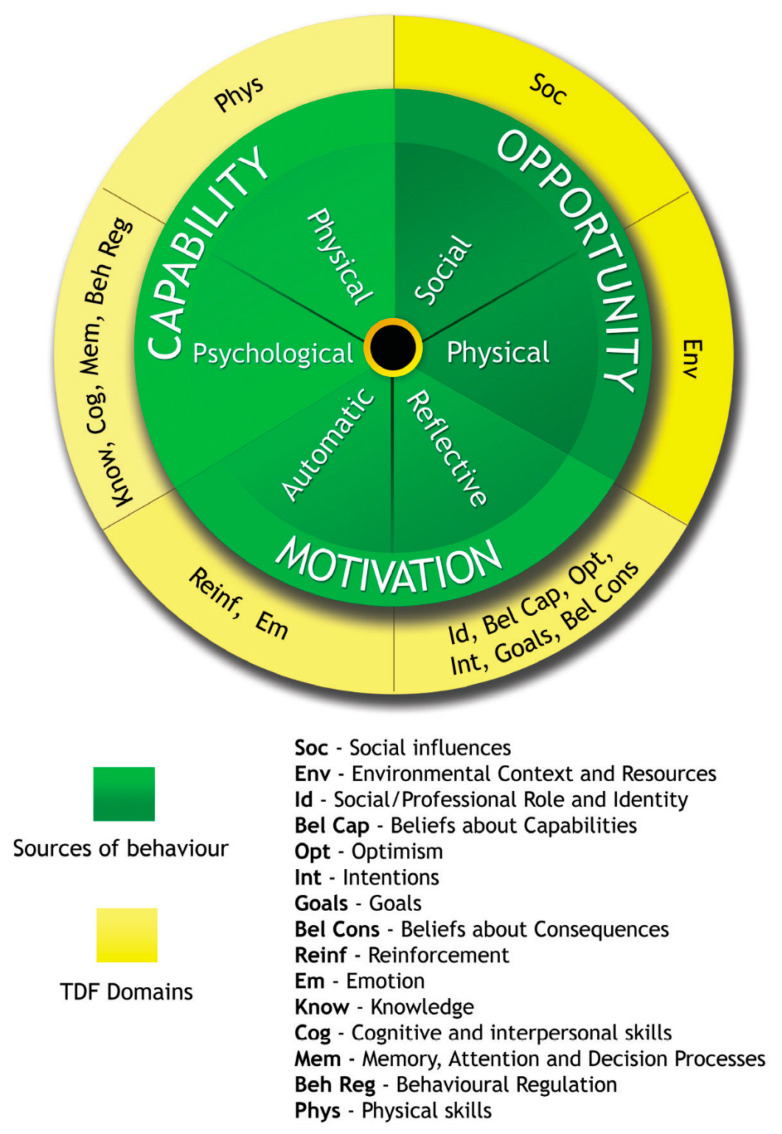
Linking the Theoretical Domains Framework (TDF) with the Capability, Opportunity, Motivation—Behaviour (COM-B) model (reprinted with permission from Ref. [25]. 2014, Michie, S.; Atkins, L.; West, R. www.behaviourchangewheel.com (accessed on 10 October 2022)).

A qualitative descriptive study of adult patients (18+ years old) with non-valvular AF was conducted utilising semi-structured individual interviews. The study’s design, execution, and reporting were informed by the Consolidated Criteria for Reporting of Qualitative Studies (COREQ) (Supplementary Material S1) [26]. TDF [23] and the COM-B model [24] underpinned the design of the interview guide and data analysis (Figure 1).

### 2.2. Participants

English-speaking patients with non-valvular AF who were aware of their AF diagnosis, eligible for oral anticoagulation based on the CHA_2_DS_2_-VASc score (a score of ≥2 in men and ≥3 in women), and had regular attendance to an Australian general practice were included in the study [3]. Non-valvular AF refers to AF in the absence of moderate to severe mitral stenosis or a mechanical heart valve [3]. We defined regular attendance as a minimum of 3 visits to the practice in the past 24 months. For the entire sample, we aimed to purposively include 1 to 2 patients who were taking warfarin and another 1 to 2 patients who were not currently taking any OACs, with the remaining patients to be anyone who fulfilled the above criteria (i.e., regardless of whether they were taking OACs or not or the specific OAC they were taking). The exclusion criteria were participants with cognitive impairment or inability to engage in verbal communication or who were unwilling or unable to provide informed consent.

### 2.3. Recruitment of General Practices and Study Participants

General practices from four states (i.e., Western Australia, New South Wales, Victoria, and Southern Australia) were approached to help identify eligible patients, with participants from two states, Western Australia and New South Wales, ultimately being represented. To determine the relative socio-economic advantage or disadvantage of where the practices were located, we used the socio-economic indexes for areas (SEIFA) score developed by the Australian Bureau of Statistics [27]. Practices were recruited through direct contact with the help of a group of GP pharmacists, i.e., pharmacists who “work directly and collaboratively with GPs and other health professionals to support the quality use of medicines” [28]. A purposive approach was followed to engage the GP pharmacists. In practices that agreed to participate, clinical audits were conducted by the GP pharmacists to help identify eligible patients. The clinical audit tool employed by the practices (i.e., PenCS CAT4^TM^ [29]) utilises the CHA_2_DS_2_-VASc score to assess stroke risk. Therefore, this score was used to identify eligible participants instead of the CHA_2_DS_2_-VA that was recommended by the Australian AF clinical practice guidelines [3]. After the audit, a list of eligible participants was identified and approached by the practices with an invitation to participate in the study using one or more of the following approaches: phone calls, emails, letters, and in-person conversations. Participants who expressed interest in participation and consented were included in the study.

### 2.4. Interview Guide

An interview guide (Supplementary Material S2) was developed (by EAG, a male pharmacist with extensive international clinical and research experience and previous experience in qualitative research) based on a review of the literature and findings from our previous qualitative study among GPs [13]. It was then refined by the research team to ensure that the questions aligned with the research objectives, target behaviour, and have clarity and good flow. As the TDF is more detailed than the COM-B and allows for a more explicit examination of influences of behaviour, the questions were informed by the 14 domains of TDF which are linked to the components of the COM-B model (Figure 1) [25].

### 2.5. Data Collection

Data were collected by one of the investigators (EAG) from March to July 2022 using semi-structured interviews. Depending on the location and preference of the participants, all interviews were conducted either face-to-face using two audio-recording devices (Philips Voice Tracer DVT5000) or online via Zoom (Zoom Video Communications, Inc., San Jose, CA, USA) using the Zoom recording function. EAG was not known to the participants before this study. Only the participant and the researcher were present during the interviews, which were 21 to 43 min long (median (interquartile range) = 36 (4.3) min). We used data saturation to determine the final sample size. Data saturation was considered to be when no new TDF domains or inductive codes emerged from the interview transcripts. This was determined by one of the researchers (EAG) with confirmation from a second researcher (KL) through the independent coding of a sub-set of transcripts and peer debriefing.

### 2.6. Data Analysis

Interviews were transcribed using online transcription software (Otter.ai, Los Altos, California, United States) and were manually checked by two of the researchers (EAG and KL) to ensure the accuracy of the transcription. We obtained formal ethics approval to utilise the online transcription software. Any errors were corrected before the transcribed data were analysed.

Sections of the interview transcripts were first coded to the 14 domains of TDF (i.e., deductively) using the NVivo software version 12 (QSR International Pty Ltd., Doncaster, Victoria, Australia) based on the framework method [30]. Before deductively coding the interview transcripts (EAG and KL), a code book that contained definitions of each TDF domain relevant to the research objectives was prepared (EAG, DD, and KL; Supplementary Material S3). Overlap between the various TDF domains was encountered while deductively coding interview transcripts to the TDF domains. In such cases, depending on the degree of overlap, coding to the domain that best fitted the data or coding to multiple domains was undertaken. Inductive coding was also applied to identify common themes within each of the TDF domains (EAG). Representative de-identified quotes were selected to exemplify the study findings. To summarise the findings, the more detailed TDF domains were mapped to the corresponding components of the COM-B model, as shown in Figure 1.

### 2.7. Quality Assurance

Various strategies were employed to ensure the quality of the study. First, the interview guide was prepared in consultation with a behaviour change expert (DD). Then, a consumer representative (AP, a patient with AF) and a second person (KS, to comment on plain language) reviewed and provided feedback on the draft interview guide. A codebook was also prepared before data coding was started. In addition, credibility, transferability, confirmability, and dependability were ensured by using analyst/investigator triangulation (initial coding completed independently by EAG and KL), negative case analysis, peer debriefing (EAG, KL, and DD), rich description, and audit–trial techniques [31,32,33,34].

### 2.8. Ethical Considerations

Prior to the recruitment of participants, data generated from the audit were kept confidential by the practices, while the researchers had access to only anonymised and aggregated data. Due to the fact that participants were approached and recruited by the practices, the researchers did not have knowledge of the participants before they consented to participate in the study. Written informed consent was sought from each study participant by the practices or GP pharmacists prior to their interview. After conducting the interviews, the collected data were anonymised with the use of pseudonyms and codes in order to maintain confidentiality. Participants were reimbursed for their time with AUD 40 gift vouchers; no reimbursement was offered for travel expenses.

## 3. Results

### 3.1. Participants

A total of 13 participants were invited and agreed to participate in the study. After excluding three participants who failed to provide written consent, ten participants from six practices took part in the semi-structured interviews, and data saturation was reached by the seventh participant. The median (interquartile range) age of the participants was 78.5 (74–81) years. The majority of the participants were male (n = 6), from Western Australia (n = 6), and had a median (interquartile range) CHA_2_DS_2_-VASc score of 3.5 (1). All of the participants were taking OACs, mainly apixaban (n = 8), while one patient had previously switched from warfarin to a DOAC. All participants were recruited from practices located in areas with relatively higher advantages, as indicated by their SEIFA scores of 4 and 5 (Table 1).

All three components of the COM-B model were identified to influence patient agreement to commence OACs recommended by their doctor. The presentation below is organised based on the components of the COM-B model and the corresponding TDF domains. Most participants reported psychological capability (i.e., knowledge), social opportunity (i.e., social influences) and reflective motivation (social/professional role or identity, goals, and beliefs about consequences) as important influences on patient agreement to commence OACs (Table 2).

### 3.2. Capability

Capability includes physical capability (e.g., physical skills) and psychological capability (e.g., knowledge or psychological skills) [25]. Participants mentioned psychological capability as an influence on patient agreement to commence OACs. Of the four TDF domains that are linked to psychological capability, knowledge was identified as important to patient agreement to commence OACs.

#### Psychological Capability

- Knowledge

***Theme: Weighing risk vs benefit.*** A clear understanding that the benefits of taking OACs exceeded the risks was identified as a facilitator of patient agreement to commence OACs by the majority of participants. On the other hand, a clear understanding of the relative advantage of apixaban over warfarin helped one participant agree to commence the recommended OAC. All but one participant mentioned bleeding as a potential side-effect of OACs, but that was not identified as a barrier to patient agreement to commence OACs.

*Well, from what I’ve been advised from the doctor etcetera, the danger obviously is possible clotting of the blood, which then can lead to either brain damage, and so on, so forth. So obviously, learning that was quite a concern at the time. And if I didn’t take it, the dangers that could develop by not taking it was enough to convince me that I was quite happy to go with it.*

*(P7)*

### 3.3. Opportunity

Opportunity includes physical opportunity (e.g., resources) and social opportunity (e.g., interpersonal influences) [25]. Both social and physical opportunity were identified as important influences of patient agreement to commence OACs.

#### 3.3.1. Physical Opportunity

- Environmental Context and Resources

***Theme: Information provision*.** The dedication of adequate time by doctors and pharmacists to provide detailed information and access to OAC-related information was reported to facilitate agreement to commence OACs by some participants.

*She [my doctor] also gave me the printout. […] I think with the verbal discussion I’ve had with the doctor, the reading I’ve done, not Google reading, information provided by the doctor written information of bits made me comfortable and quite happy to go into the program with apixaban.*

*(P7)*

#### 3.3.2. Social Opportunity

- Social influences

***Theme: Explanations from doctors.*** Some participants reported explanations from doctors as a facilitator to patient agreement to commence OACs. On the other hand, reportedly insufficient explanations could be a potential barrier to commencing the OAC recommended by doctors, as explained by one participant.

*I’ve never taken anything that has not been fully explained to me and which I have agreed to, as far as the explanation is concerned to accept it. […] And I was explained to it why [I should take apixaban].*

*(P1)*

***Theme: Relationship with doctors:*** Having good relationships with doctors was a prominent facilitator for almost all participants. Participants reported that, because they trust their doctors, they agree with recommendations from their doctors regarding their medication-related needs, including OACs.

*I’m reasonably confident in the apixaban because I trust the doctor that I see. I have to trust them. It’s pointless going if you don’t trust them. *

*(P6)*

***Theme: Other people’s experiences or views.*** Other people’s experiences with or views on OACs were reported to be potential facilitators or barriers. Two participants, while acknowledging that other people’s experiences or views do not necessarily affect their decision because other people’s cases are different from theirs, reported that being aware of the benefits of OACs from other people they knew helped them ‘make sense’ when they were recommended by their doctors. On the other hand, one participant reported that he was concerned about OACs because of the experiences of other people until it was explained to him by his doctor. A concern about the inconvenience of taking warfarin based on the experiences of other people was also reported by one participant.

*I’d heard of the benefits, I suppose. And I knew at least two other people that were taking the same sort of thing. So, it [taking an OAC] just made sense to me. *

*(P5)*

*I was concerned when my friends having this stuff [blood thinners] that they had to take. But when it is explained why I had to take it, I said, that’s the story these guys got and that’s why they’re taking it. And that’s why they’re still here.*

*(P7)*

### 3.4. Motivation

Motivation includes reflective (e.g., beliefs about what is good or bad) and automatic motivation (e.g., reflex responses) [25]. Both automatic and reflective motivation were important factors affecting patient agreement to commence OACs. Almost all TDF domains linked to these components of the COM-B model, especially those corresponding to reflective motivation, were identified as important influencers.

#### 3.4.1. Reflective Motivation

- Social or professional role and identity

***Theme: Passive role in the decision-making.*** While some participants reported potentially seeking information about their medications, most of them described themselves as compliant individuals who follow their doctors’ advice, and hence, agreed to commence the recommended OAC. Some participants believed that their limited health literacy contributed to their willingness to start OACs upon their doctor’s recommendation. Three participants specifically acknowledged their lack of knowledge about health and medications, relying on doctors for their medication needs.

*As it was advised by my doctor, I wouldn’t have a reason to refuse to take it. […] I don’t question. The doctor knows what they’re doing.*

*(P9)*

b. Goals

***Theme: Preventing possible danger.*** A prominent facilitator to patient agreement to commence OACs for most of the participants was its alignment with patients’ broader goals of preventing possible future danger including intellectual and physical limitations due to stroke-associated disability.

*I still want to do a lot of things. And the stroke would sort of wipe me out, I’m sure, something from doing lots of things. I don’t want that. *

*(P2)*

***Theme: Commencing OACs while aiming to reduce pill burden.*** Some participants reported that they aim to take as few medicines as possible in the management of medical conditions. One participant expressed concern with and a desire to reduce the higher number of pills he was taking.

*I don’t have a problem taking medication. What I do have a problem with is taking too much medication. I would like to cut back on a lot of this medication. […] And if I can reduce it without having any side effects, then I would do it.*

*(P6)*

c. Intentions

***Theme: Openness to agreeing with doctors’ recommendations.*** Most participants showed openness to agreeing to commence OACs that were recommended by their doctors. This was especially true if adequate explanations were given and if their doctors’ recommendations aligned with their beliefs. More specifically, two participants showed openness to taking warfarin despite recognising potential side-effects and other disadvantages associated with taking warfarin.

*I’m a great believer in the medical association. If they tell me to do something, with one or few exceptions, I tend to subscribe to it. So, when they told me to put me on the blood thinners, I presumed there was a good reason. And I’ve been using them ever since. *

*(P6)*

d. Beliefs about consequences

***Theme: OACs and stroke prevention.*** Expected benefits of stroke/clot prevention from taking OACs were identified to influence patient decision-making. Most participants reported expecting OACs to prevent stroke and acceptable bleeding risk as facilitators to patient agreement to commence OACs.

*P5: So, I was quite happy to have blood thinners which would save that problem, less likely to have a stroke. […] Stop a blood clot going to my brain or my lungs.*

e. Optimism

***Theme: Confidence regarding efficacy and/or safety of OACs.*** The confidence that participants had regarding the stroke/clot prevention and bleeding effects of OACs constituted an important motivation for patient agreement to commence an OAC for most participants.

*Well, just reassuring that we do have a medicine such as apixaban to help prevent strokes being proven, I think to help prevent strokes. So, I feel very confident taking it.*

*(P8)*

#### 3.4.2. Automatic Motivation

- Reinforcement

***Theme: Previous experience.*** Previous experience was a potential facilitator to patient agreement to commence OACs. Three participants expressed previous positive experiences with their doctors or their medications, including OACs, as positive reinforcements to their agreement to commence the recommended OACs.

*I don’t think I would refuse if my doctor said this is the best thing for you. I’ve got faith in the medical profession and my specialists. And up to now, all the doctors I’ve seen have done the right thing by me. *

*(P2)*

One participant reported mixed thoughts when switched from warfarin to rivaroxaban.

## 4. Discussion

### 4.1. Statement of Key Findings

This qualitative study aimed to explore the perspectives of patients with AF regarding barriers and facilitators to patient agreement to commence OACs. The COM-B model and TDF were used to systematically categorise and identify key barriers and facilitators. We found that reflective motivation, psychological capability, and physical and social opportunity were important facilitators to patient agreement to commence OACs. On the other hand, social opportunity and reflective motivation were identified as potential barriers.

### 4.2. Interpretation

Almost all patients reported passive roles in decision-making and openness to agreeing with recommendations from their doctors, which were identified as facilitators. This was in line with a previous qualitative study wherein GPs reported that most patients agree with the recommendations from their doctors [13]. The passive roles of participants may also be attributed to their advanced age, as earlier studies have indicated that older patients tend to rely on their doctors for the prescription of new medications [35]. Moreover, some of the participants in this study reported perceived low health literacy, which made them rely completely on their doctor’s recommendations. However, reportedly inadequate understanding of the AF and OACs may have potential negative implications on long-term OAC management such as medication adherence [36,37]. Hence, efforts should be made to improve patients’ understanding of AF, OACs, and thromboprophylaxis during and after OAC initiation.

Despite this, the alignment of doctors’ recommendations with patients’ overall health goals, including prevention of stroke and associated physical and intellectual disabilities, appeared to facilitate patient agreement to commence OACs. Previous studies have reported that concerns about stroke risk associated with AF can encourage patients to be actively involved in their own healthcare, such as through participation in AF screening programs and agreeing to commence antithrombotic therapy for stoke prevention [38,39]. Therefore, clearly articulating that taking an OAC can help reduce stroke risk can motivate patients to agree to commence an OAC with an informed perspective.

While most participants reported adopting passive roles, failing to involve patients as active partners in the decision-making process could be a barrier to patients’ agreement to commence OACs. On the other hand, when they were provided with adequate explanations and had a clear understanding, participants reported willingness to accept the bleeding risk of OACs in order to prevent stroke. This is also in line with previous studies; patients were willing to accept higher bleeding risks for the sake of preventing stroke, especially if adequate explanations were provided [13,40]. Additionally, doctors’ explanations reportedly had a positive influence in terms of changing the negative perception patients had of OACs based on the experiences of friends. This is important, as existing literature highlighted the impact of friends and family members’ experiences on patients’ decision-making [41]. Offering comprehensive explanations and engaging patients as active partners in a collaborative decision-making process, taking into account their beliefs, values, and preferences, aligns with the recommendations outlined in the AF guidelines [3].

Having trusting relationships with doctors was an important facilitator, as patients who have good rapport with their GP are likely to trust their judgement. This may have contributed to patients’ reported passive roles in decision-making and their openness to agreeing with recommendations from their doctors. In a previous study, GPs reported that patients were more likely to agree with doctors’ recommendations if a trusting relationship has been established, such as the patient being cared for by a regular doctor [13]. Furthermore, previous positive experience with either doctors or medications (including OACs), friends’ or family members’ experiences with OACs, access to OAC-related reading materials, and adequate time for explanations reportedly motivated patients to agree to commence the recommended OACs.

In view of the influences of patients’ agreement to commence OACs discussed above, interventions incorporating patient education that take patients’ overall health goals and beliefs into consideration, such as adequate explanations and reading materials on AF-related stroke risk and the benefits of OACs, would be useful. Supplementing such interventions with the use of patient decision aids such as imagery, videos, and figures may also be helpful in reassuring patients of the advantages of agreeing to commence OACs. Additionally, a previous systematic review suggested that interventions to improve guideline adherence in patients with AF in the primary care setting were more effective when they were multifaceted [42]. Interventions that target behaviour change are likely to be more effective [24], but they need to be evaluated by future research. Based on these insights, multifaceted interventions may be considered to encompass various components, including patient-centred AF education, involving family members and caregivers, empowering patients to make informed decisions through the provision of written materials, and utilising patient shared-decision aids, such as videos [43,44].

## 5. Limitations

All of the participants in this study were individuals currently prescribed OACs, which may have resulted in underrepresentation of possible barriers to patient agreement to commence OACs. Additionally, all participants were taking DOACs, but not warfarin. However, more than three-quarters of patients with AF attending Australian primary care take DOACs, and the trend is growing [4]. Second, despite operational definitions of each domain being prepared in line with research objectives, coding interview transcripts into TDF domains revealed apparent overlaps. This limitation of the TDF framework was observed, echoing challenges faced by earlier studies in coding to specific domains [45,46]. Recall bias was a potential concern, and future studies focusing on newly diagnosed or OAC-prescribed patients with AF could mitigate this issue. It is also essential to acknowledge the restricted age range of the participants in this study. Furthermore, participant recruitment was limited to urban areas in two states, and the inclusion of GP pharmacists and practices in SEIFA categories 4 and 5 indicates a non-representative sample of all Australian GP practices. It is worth noting that no data on participants’ ethnicity were collected, which could impact the representativeness of the sample. This limited diversity may have contributed to data saturation being reached relatively quickly, potentially affecting the richness of the insights obtained. Lastly, many reported positive views towards their health providers and medications, which may not accurately reflect the perspectives of those who opted not to commence OACs. Nevertheless, this study shed light on patient perspectives regarding OAC initiation in the Australian general practice setting.

## 6. Conclusions

Although most patient participants in this exploratory study reported passive roles in decision-making, other prominent factors—including the alignment of recommendations with patients’ overall health goals, adequacy of explanations principally from doctors, and a clear understanding of the advantages and disadvantages of taking OACs—influence whether they agree to commence OACs. Therefore, targeting influences on patient agreement to commence OACs should be an integral part of any quality improvement interventions. Future studies should also explore the perspectives of OAC-eligible patients with AF who do not agree to commence OACs. Investigating influences on continuing to take OACs from the perspectives of patients and doctors should also be a focus of future research.

## Figures and Tables

**Table 1:** Participants included in this study.

ID	State	SEIFA Score (Practices)	Sex	Age	CHA2DS2-VASc Score	Time Since Diagnosis of AF (Patient-Reported)	Current Antithrombotic Medication ^a^	Setting of Data Collection
P1	WA	4	M	81	4	0–4 years	Apixaban	In-person
P2	NSW	5	M	78	4	0–4 years	Apixaban	Zoom
P3	WA	4	M	74	5	5–10 years	Apixaban and Aspirin	In-person
P4	WA	4	M	99	3	≥16 years	Apixaban	In-person
P5	WA	4	F	72	3	0–4 years	Rivaroxaban	In-person
P6	NSW	5	M	77	4	5–10 years	Apixaban	Zoom
P7	WA	4	M	81	3	0–4 years	Apixaban	In-person
P8	WA	4	F	79	3	0–4 years	Apixaban	In-person
P9	NSW	5	F	74	3	11–15 years	Apixaban ^b^	Zoom
P10	NSW	5	F	85	4	5–10 years	Rivaroxaban (switched from warfarin)	Zoom

^a^ Obtained from medical notes as reported by the GP pharmacist and confirmed by the patient; ^b^ GP pharmacist reported dabigatran, but patient reported apixaban; NSW: New South Wales; WA: Western Australia.

**Table 2:** Themes aligned with the COM-B and TDF domains.

COM-B Domain	COM-B Sub-Domain	TDF Domain	Themes
Capability	Psychological capability	Knowledge	Weighing risk vs benefit.
Opportunity	Physical opportunity	Environmental context and resources	Information provision.
	Social opportunity	Social influences	Explanations from doctors.
			Relationship with doctors.
			Other people’s experiences or views.
Motivation	Reflective motivation	Social or professional role and identity	Passive role in the decision-making.
		Goals	Preventing possible danger.
			Commencing OACs while aiming to reduce pill burden.
		Intentions	Openness to agreeing with doctors’ recommendations.
		Beliefs about consequences	OACs and stroke prevention.
		Optimism	Confidence regarding efficacy and/or safety of OACs.
	Automatic motivation	Reinforcement	Previous experience

## Data Availability

The data that support the findings of this study are available on request from the corresponding author. The data are not publicly available due to ethical restrictions.

## Supplementary Materials

The following supporting information can be downloaded at: https://www.mdpi.com/article/10.3390/pharmacy11050153/s1, File S1: COREQ checklist; File S2: Interview guide; File S3: Coding book.
