# Supplementary material for: Patients’ Perspectives on Commencing Oral Anticoagulants in Atrial Fibrillation: An Exploratory Qualitative Descriptive Study
Source: Pharmacy (Basel). 2023 Sep 23;11(5):153. doi: 10.3390/pharmacy11050153 (PMC10609834; doi:10.3390/pharmacy11050153)
Supplement: Supplementary file 1 [file pharmacy-11-00153-s001.zip › Supplementary material S2-Interview guide_.pdf]

Appendix 2: Interview guide

1. When were you first diagnosed with atrial fibrillation?
   - *Follow-up:* What is your understanding of what atrial fibrillation is? (*Prompt*: symptoms, causes, risks, management…)
   - *Follow-up:* Have you had any problems because of your atrial fibrillation?
     - *Prompt:* What does living with atrial fibrillation mean or look like to you?
     - *Follow-up:* What (other) problems which are associated with atrial fibrillation do you know?
     - *Prompt:* Are you aware of any other ways atrial fibrillation could affect you?
   - *Follow-up:* If mentioned – What are your concerns around the stroke risk associated with your atrial fibrillation?
   - *Follow-up:* If mentioned – How do you think you can prevent stroke because of atrial fibrillation?
2. What do you know about oral anticoagulant (blood thinner) medications?
   - *Follow up:* Are you currently on any blood thinner medication?
     - *Follow-up:* If yes –Which blood thinner medication? Why do you take this medication?
     - *Follow-up*: If no – Has your GP or any other prescriber suggested that you should take one?
     - *Follow-up*: If aspirin or any antiplatelet mentioned – Has your GP or any other prescriber suggested that you should take it?
   - *Follow up:* What other types of blood thinner medications do you know about?
     - *Prompt*: warfarin, apixaban (Eliquis), rivaroxaban (Xarelto), and dabigatran (Pradaxa) – May be show the packages
   - *Follow up:* If currently not taking a blood thinner – Do you plan to take a blood thinner medication in the near future (if prescribed by your doctor)?
   - *Follow up:* If already taking a blood thinner – What reasons would make you refuse/be reluctant to take a blood thinner medication that was recommended by your doctor?
   - *Follow-up*: If refused/ reluctant – What would you need for you to decide that taking a blood thinner medication was right for you?[#]
   - How do you think things would go if you accepted a prescription for a blood thinner?

3. What is the first thing that comes to your mind when you hear the term ‘an oral anticoagulant’ or ‘a blood thinner’?
    - *Follow-up:* How do you see the benefits of blood thinner medications in the management of your atrial fibrillation?
    - *Follow-up:* What side effects do you expect from being on a blood thinner medication?
4. What do you typically do when your doctor prescribes a new medication for you? Do you instantly agree to start taking the medication as prescribed?
    - *Follow-up:* How about when it comes to taking a blood thinner medication that is/if recommended by your GP? [#]
5. To what extent is taking a blood thinner medication a priority for you?
    - *Prompt:* How important is taking a blood thinner medication to you?
    - *Follow-up:* What makes you say that?
6. What made you decide to take/not to take a blood thinner medication?
    - *Follow-up:* Before making the decision to take/not to take your blood thinner medication, how adequate were the explanations from your GP?
        - *Follow-up:* What makes you say that?
    - *Follow-up*: Who made the decision on whether you should/should not be taking a blood thinner medication? Prompt: Your GP, yourself, both, or others?
    - *Follow-up*: How confident do you feel about weighing up the pros and cons of taking a blood thinner medication for your atrial fibrillation?
        - *Prompt*: Do you feel able to weigh up the pros and cons of taking a blood thinner medication for your atrial fibrillation?
    - *Follow-up*: How would the views/opinions of others (e.g. family, friends, other patients....) affect your decision to take the blood thinner medication that was recommended by your GP?
    - *Follow-up*: How would your or other people’s previous experiences with blood thinner medications affect your decision to take/ not to take a blood thinner medication?
7. How confident are you that taking your blood thinner medication will make a difference in your health?
    - *Follow-up:* What makes you say that?

- *Follow-up*: Do you feel you have the necessary support/help available to take your blood thinner medication as prescribed? (Prompt: family, friends, GP, specialist…)

8. What other comments do you have on taking a blood thinner medication for your atrial fibrillation?

Thanks for your time and your input. You have been very helpful.
