# Supplementary material for: Patients’ Perspectives on Commencing Oral Anticoagulants in Atrial Fibrillation: An Exploratory Qualitative Descriptive Study
Source: Pharmacy (Basel). 2023 Sep 23;11(5):153. doi: 10.3390/pharmacy11050153 (PMC10609834; doi:10.3390/pharmacy11050153)
Supplement: Supplementary file 1 [file pharmacy-11-00153-s001.zip › Supplementary material S3_Coding book_.pdf]

TDF domains	Operational definitions
**Knowledge** (An awareness of existence of something)	• Awareness that AF increases the risk of blood clotting and/or stroke risk.<br>• Awareness and knowledge of what OACs are.<br>• Knowledge of how OACs work and can be helpful
**Beliefs about Consequences** (Acceptance of the truth, reality, or validity about outcomes of a behaviour in a given situation)	• What the participant expects/ed from being on an OAC.<br>• Believing/expecting that OACs help reduce the participant's risk of blood clots and/or stroke.<br>• Expecting bleeding from being on an OAC.
**Optimism** (The confidence that things will happen for the best or that desired goals will be attained)	• The confidence that the OAC the participant is taking will protect the participant from experiencing a blood clot/stroke.<br>• The confidence that the OAC the participant is taking will/will not cause serious bleeding.
**Emotion** (A complex reaction pattern, involving experiential, behavioural, and physiological elements, by which the individual attempts to deal with a personally significant matter or event)	• Participant's feeling about accepting/refusing to take an OAC
**Skills** (An ability or proficiency acquired through practice)	• Participant's communication/interpersonal skills with doctors, other healthcare professionals, and other people with regard to AF and OAC management.
**Beliefs about capabilities** (Acceptance of the truth, reality or validity about an ability, talent or facility that a person can put to constructive use)	• Participant's belief in one's capability to accept an OAC recommended by the doctors.<br>• Participant's belief in one's ability to self-monitor.<br>• Participant's belief in one's ability to recognise the need to seek help.
**Reinforcement** (Increasing the probability of a response by arranging a dependent relationship, or contingency, between the response and a given stimulus)	• Increased/decreased probability of accepting OACs because of participants' previous self-experience with current OAC or other OACs/antiplatelet agents.<br>• Participant's perceived reinforcement from the doctor if accepting the recommended medication
**Goals** (Mental representations of outcomes or end states that an individual wants to achieve)	• The extent to which accepting to take an OAC compliments with patients broader health goals.
**Intentions** (A conscious decision to perform a behaviour or a resolve to act in a certain way)	• Participant's intention to accept an OAC prescribed by a doctor.<br>• Participant's intention to follow advice from the doctor.

**Memory, attention and decision processes** (The ability to retain information, focus selectively on aspects of the environment and choose between two or more alternatives)	• Participant's ability to understand and retain AF- and OAC-related information in accepting/not accepting to take the recommended OAC.<br>• Participant's ability to choose a specific OAC from two or more alternatives.<br>• Participant's ability to weigh the risk vs benefit of taking OACs.
**Social/professional role and identity** (A coherent set of behaviours and displayed personal qualities of an individual in a social or work setting)	• Participant's perceived role in accepting to take the recommended OAC.<br>• Believing that patients should do as told without asking questions.
**Social influences** (Those interpersonal processes that can cause individuals to change their thoughts, feelings, or behaviours)	• The influence of other people's experience with OACs on participant's acceptance an OAC recommended by a doctor.<br>• The influence of other people's opinions on OACs on participant accepting an OAC recommended by a doctor.<br>• Availability of support from people (family, friends, healthcare professionals, etc.) that helped the patient accept to take OAC as recommended.<br>• Influence of participant's relationship with the doctor on accepting OACs or other medications recommended by the doctor.
**Environmental context and resources** (Any circumstance of a person's situation or environment that discourages or encourages the development of skills and abilities, independence, social competence and adaptive behaviour)	• Barriers and facilitators (e.g. the healthcare system, distance from various health services, cost, etc.) that affect participant's acceptance of taking the recommended OAC.
**Behavioural regulation** (Anything aimed at managing or changing objectively observed or measured actions)	• Participant's ability to regulate acceptance of different medications.
